# Utilization of Wind Turbine Blade Waste in the Production of ABS Composites and Selected Products Based on These Composites

**DOI:** 10.3390/polym17060796

**Published:** 2025-03-17

**Authors:** Rafał Malinowski, Volodymyr Krasinskyi, Krzysztof Bajer, Oksana Krasinska, Piotr Augustyn, Anna Pietruszka, Krzysztof Moraczewski

**Affiliations:** 1 Łukasiewicz Research Network—Institute for Engineering of Polymer Materials and Dyes, 55 M. Skłodowska-Curie St., 87-100 Toruń, Poland; volodymyr.krasinskyi@impib.lukasiewicz.gov.pl (V.K.); krzysztof.bajer@impib.lukasiewicz.gov.pl (K.B.); oksana.krasinska@impib.lukasiewicz.gov.pl (O.K.); 2 Faculty of Materials Engineering, Kazimierz Wielki University, 30 Chodkiewicza St., 85-064 Bydgoszcz, Poland; augustyn@ukw.edu.pl (P.A.); kmm@ukw.edu.pl (K.M.); 3 Łukasiewicz Research Network—Institute of Heavy Organic Synthesis “Blachownia”, 9 Energetyków St., 47-225 Kędzierzyn-Koźle, Poland; anna.pietruszka@poczta.onet.pl

**Keywords:** wind turbine blades, mechanical recycling, ABS composite, glass fibers, extrusion, mechanical properties

## Abstract

The paper presents studies on the use of waste from wind turbine blades (WTBs) in the production of thermoplastic composites and regranulate-based products of acrylonitrile-butadiene-styrene (ABS) copolymers. Composites containing two types of WTB fractions (finely milled fraction—GRm and dust fraction—GRd) were produced using a co-rotating twin-screw extruder. During extrusion, different screw configurations of the plasticizing system as well as different material formulations were investigated. The studied composites contained from 10 to 70 wt% of shredded WTB, as well as up to 15 wt% of additional components, mainly those improving impact strength and processing properties. It was found that the individual WTB fractions generally deteriorate the mechanical properties of ABS. However, a composite containing 30 wt% GRm and modified with an additional 7 wt% ACM-G2 (impact modifier type) can be hot-pressed into good quality panels. It can also be successfully used to produce profiles in the extrusion process, mainly due to its significantly reduced viscosity. The studies presented in this article showed one of the possible ways of using WTB waste. It is advantageous because it uses WTB waste in a thermoplastic ABS matrix, which is also a secondary raw material. As a consequence of this, a completely new composite material based wholly on secondary raw materials can be obtained, which can be subjected to multiple processing.

## 1. Introduction

Wind power plants are a clean, renewable, inexhaustible and efficient source of energy. Currently, the global amount of energy obtained from this type of source is dynamically increasing (540 GW in 2017, 650 GW in 2019, 930 GW in 2022) and currently exceeds the value of 1 TW [1]. Along with this increase, there is also a growing demand for more modern and more efficient wind turbines that generate more and more power. The largest and most common group among them is Horizontal Axis Wind Turbine (HAWT). However, the rapid development of the HAWT market leads to a situation in which, on the one hand, more and more modern clean energy turbines are produced, and on the other hand, significant amounts of new materials are created, which should be recycled or disposed of at the end of their useful life. This applies mainly to wind turbine blades (WTBs), which after a certain period of use, usually after 20–25 years, are decommissioned due to ageing processes or mechanical damage, including the formation of delaminations [2,3]. The number of withdrawn WTBs is expected to multiply in the next 5–10 years depending on the installed power of the turbines. It is also estimated that after 2035, at least 225,000 tons of WTB will have to be recycled worldwide [4]. Other data indicate that the amount of waste from WTB will increase from ca. 400,000 tons in 2030 to ca. 1 million tons in 2050 [5]. Regardless of the quantitative data, WTB recycling technologies will have to develop rapidly, especially if WTBs are made of newer and more complex materials.

The traditional wind turbine blades are mostly made of resins reinforced with fibers, such as glass (GF), basalt (BF), carbon (CF) or hybrid GF/CF [6,7]. Moreover, in recent years, WTBs are being increasingly made of many different materials, which leads to a situation in which multi-material waste generated from them is difficult to recycle. This is the reason why some of them are stored in landfills. The WTB waste constitutes, therefore, a significant environmental protection problem, which may deepen in the coming years due to increased WTB production. For environmental protection and sustainable development, the best way to deal with WTB waste is to recycle and reuse it, so that wind energy can become a real “clean energy”. Therefore, new, efficient and economical technologies for recycling are constantly being sought.

Many efforts are currently underway to use different types of recycling to reuse WTB waste [8,9,10]. This applies to mechanical recycling [9,11,12], thermal recycling [13] or chemical recycling [14], or even hybrid (thermochemical) recycling [15]. In mechanical recycling, WTB waste is shredded and fractionated into different particle sizes, which can be used as a filler in polymeric materials or as a reinforcing phase for other materials, e.g., ceramics. The main disadvantage of this process is the significant destruction of the fiber structure and the inability to accurately separate the individual phases (matrix and reinforcing phase). The mechanical properties of the fiber are significantly reduced, and the potential use of the recycled product depends mainly on the size of the particles obtained. Pure fibers can be recovered in thermal or chemical recycling. The use of the former is simpler, but the gases generated, which are often toxic, can be easily emitted, and the resulting ashes generate secondary environmental pollution. In addition, heat-treated fibers are destroyed and lose their good mechanical properties. Various varieties of this recycling, mainly pyrolysis [16,17], microwave pyrolysis [18,19], plasma technology [20] or the fluidized bed recycling process [21], have been widely presented in the literature reports [18,22,23,24]. On the other hand, the use of chemical recycling is generally the most technologically complex and economically less advantageous process, but the fibers obtained have good functional properties. Chemical recycling (carried out mainly by the supercritical fluid method [25,26,27], the solvolysis method [28,29,30] or the electrochemical method [31]) of composites based on resins and various types of fibers used, i.e., for the production of WTB, is the subject of numerous research papers, the results of which have been widely published [32,33,34,35]. In general, different products (fibers, chemical compounds including monomers or oligomers) with different properties can be recovered by different recycling methods. This allows recovered products to be reused in various ways [36]. Directly recovered products, such as fibers, are used to make new composite materials. They can also be used in modifying building materials such as cement, concrete or polymer mortar. It is also worth mentioning the technology of co-processing WTB waste in cement production (cement co-processing), which is currently the most common technology for recycling composite waste generated by the wind industry [37]. Compared to this technology, the other technologies presented above are less price-competitive or conducted on a smaller scale. Therefore, the most favorable technological solutions are constantly being sought in order to make the best possible use of WTB after the EOL (end-of-life) service life.

In this work, we focus on the issues of the mechanical recycling of WTBs, which are the subject of our study. In recent years, many papers have been published on these issues. For example, Jensen and Skelton [38] presented studies on WTB shredding using jaw knives in one of the first and basic stages of WTB mechanical recycling. Crushers, mills or shredders are also used for shredding [39]. Crushing or shredding leads to the formation of small fragments, which are then ground into very fine particles that are fractionated. Schmidl and Hinrichs [40] recycled rotor blades made of GF-reinforced thermoset composites by mechanical recycling, which were then used for cement production. In turn, Rahimizadeh et al. [12] proposed the use of the mechanical recycling of WTBs to obtain a product used in 3D printing as a reinforcement phase for printed products.

Mechanically recycled WTB products have been used in various areas. Shredded or crushed WTB was used, for example, in particleboard and in paints and varnishes to protect wood from UV radiation [38]. Shredded WTB fractions were also used as a reinforcing phase in polymers, with the finest fractions also used to modify the properties of PLA filaments [41]. Some WTB fractions were used to produce hybrid composites with natural fibers, such as pine wood [42]. Shredded WTB also found applications in the production of railroad sleepers, subway sleepers, yard rails, jersey barriers, bollards, and utility poles [43]. WTB waste also found widespread use in the construction industry, mainly as a reinforcing phase for concrete [44,45,46]. Other applications of WTB waste include acoustic barriers made of composite resins [47], resin sheets or resin-based floor tiles [48], and asphalt mixtures reinforced with fine fractions of ground WTB [49]. Shredded and milled WTB can also be used in geopolymer composites [46]. Applications in architectural objects, road construction, and in the production of geotechnical blocks, floating platforms, and observation towers are also popular [50]. In these applications, two main directions of WTB waste utilization can be observed. The first relates to the use of various milled WTB fractions in resin composites, most often based on the same non-thermoplastic polymer matrix. The second direction is related to the use of this type of waste in construction, mainly in modifying the properties of concrete or asphalt.

Our studies focused on the use of shredded WTB fractions to produce thermoplastic composites designed for the manufacture of thick sheets or panels by hot-pressing, as well as for the production of extruded profiles. The main objectives of our studies were: (i) attempt to use suitably shredded WTB fractions to produce thermoplastic composites on an acrylonitrile-butadiene-styrene (ABS) copolymer matrix in the twin-screw extrusion process, (ii) determine the properties of the composites obtained, and (iii) manufacture finished products from the previously developed composites in the form of a suitably thick panel (by hot-pressing) or a profile (by extrusion). Changes in structure, mass flow rate, and mechanical properties of the produced materials, upon the addition of various amounts of WTB fractions and other additives, were determined. The results of these studies are presented in this publication. This is a novel issue because it refers to a dual approach to recycling. On the one hand, WTB waste is utilized, and on the other hand, the ABS we use is also from recycled waste, specifically, electro-waste. The utilization of these two wastes should significantly improve the natural environment, i.e., significantly reduce the load on the environment by waste originating from used WTB and electro-waste.

## 2. Methodology

### 2.1. Materials

The following materials were used in the investigations:

- Acrylonitrile-butadiene-styrene copolymer (ABS), type ER ABS 9004 standard 8 (Elektro Recykling, Nowy Tomysl, Poland). Its melt flow rate (MFR) was 22 g/10 min (10 kg, 220 °C), density 1.03 g/cm^3^, and impact strength 8 kJ/m^2^. It was regranulate from the disposal of electronic devices.
- Styrene-butadiene-styrene copolymer (SBS), type KRATON™ D0243 E Polymer (Kraton Polymers Nederland B. V., Amsterdam, The Netherlands) with bound styrene of 31% by mass, hardness 70 (Shore A, 10 s).
- Styrene-isoprene-styrene copolymer (SIS), type KRATON™ D1161 P Polymer (Kraton Corporation, Houston, TX, USA) with polystyrene content of 15% by mass, hardness 32 (Shore A, 10 s).
- Chlorinated polyethylene (CPE), type CPE-135C (Shandong Rike Chemical Co., Ltd., Weifang, China). Its bulk density was 0.5 g/cm^3^, chlorine content, 35%, hardness, 65 (Shore A, 10 s).
- Impact modifier (I) (ACM), type ACM-G2 (MAJUMI CHEMICALS, Radom, Poland) with bulk density of 0.5 g/cm^3^. ACM was a grafted copolymer of light-chlorinated HDPE and acrylate with structure of CPE-grafted-polyacrylate.
- Impact modifier (II) (SC), type SCONA TPKD 8304 PCC (BYK-Chemie GmbH, Wesel, Germany) with a melt volume rate (MVR) of 5–15 cm^3^/10 min (5 kg, 230 °C). SC was an impact modifier based on thermoplastic elastomer styrene-ethylene-butylene-styrene (SEBS) functionalized with glycidyl methacrylate (30% styrene content).
- Processing and mechanical properties modifier (BYK), type BYK-MAX P 4101 (BYK-Chemie GmbH, Wesel, Germany) with bulk density of 0.53 g/cm^3^. BYK was an additive based on a fatty acid ester.

ABS was used as a polymeric thermoplastic matrix. Other materials (SBS, SIS, CPE, ACM, SC and BYK) were used as modifiers of the properties of the composites produced. They were mainly aimed at enhancing the dynamic impact strength of the composites obtained as well as improving their processing properties.

**Figure 1:**
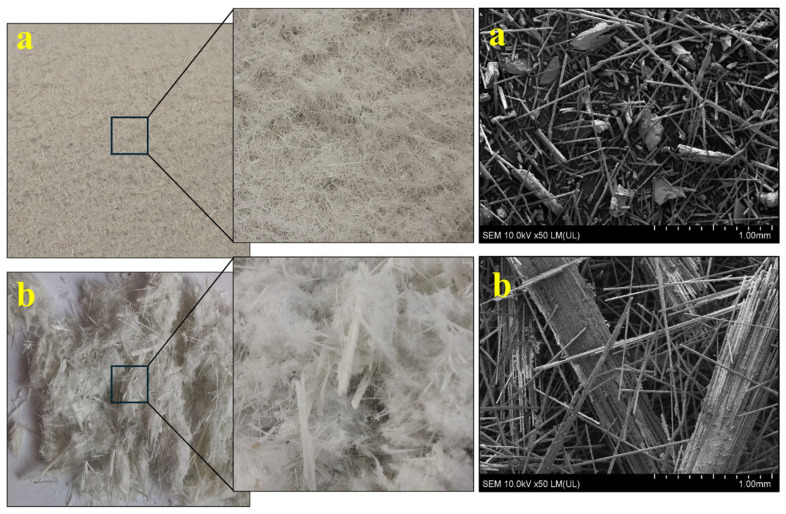
Images of shredded WTB fractions: (**a**) GRd, (**b**) GRm.

Two fractions of shredded WTB (GR) with a particle size of less than 1 mm (dust, GRd) and from 1 to 7 mm (finely milled fraction, GRm) were used in the study (Figure 1). Both fractions contained 46–49% and 47–51% alkyd resin matrix and 47–50% and 46–51% glass fibers, respectively. They also contained negligible amounts of moisture at 0.3% (GRd) and 0.5–0.6% (GRm). Furthermore, both fractions were characterized by good thermal stability; the onset of thermal decomposition (T_d5_) of the resin matrix started at a temperature of ca. 292 °C (T_d5_—the temperature at which 5% mass loss occurs).

### 2.2. Apparatus

The following devices were utilized to prepare and investigate the studied composites:

- Co-rotating twin screw extruder, type BTSK 20/40D (Bühler, Braunschweig, Germany), equipped with screws 20 mm in diameter and an L/D ratio of 40, designed for the production of granulated PCL composites.
- Screw injection molding machine, type Battenfeld Plus 35/75 (Battenfeld GmbH, Solingen, Germany), equipped with a screw 22 mm in diameter and an L/D ratio of 17, intended for the production of standard dumbbell- and bar-shaped specimens.
- Scanning electron microscope, type Hitachi SU8010 (Hitachi, Tokyo, Japan), designed to examine the geometrical surface structure of sample fractures as well as the adhesion at the phase boundary of components.
- Capillary plastometer, type LMI 4003 (Dynisco, Franklin, TN, USA), designed to determine the mass melt flow rate.
- Pendulum Impact Tester, type IMPats-15 (ATS FAAR, (ATS FAAR, Novegro-Tregarezzo, Italy), intended to determine Charpy impact strength.
- Tensile testing machine, type TIRAtest 27025 (TIRA Maschinenbau GmbH, Schalkau, Germany), designed to examine mechanical properties under static tension and static three-point bending.
- Helium pycnometer, type Ultrapycnometer 1000 (Quantachrome Instruments, Boynton Beach, FL, USA), designed for specific density determination.
- Shore hardness tester, type D (Zwick, Ulm, Germany) designed to determine changes in the composite hardness.
- MAX 50/1 moisture analyzer (RADWAG, Radom, Poland), intended for determination of moisture content.
- FTA II tester (Rheometric Scientific Ltd., Piscataway, NJ, USA), designed to determine ignitability by the oxygen index method.
- A device designed to measure resistivity, composed of a ring electrode system, model 8009 (Keithley, Cleveland, OH, USA) and an electrometer, model 6517A (Keithley, Cleveland, OH, USA).
- Thermogravimetric analyzer, type Q500 (TA Instruments, New Castle, DE, USA), designed to determine the thermal stability of studied composites.
- Hydraulic press, type LP-S-50 (LabTech Engineering, Samut Prakan, Thailand), designed for the manufacture of panels with dimensions of 300 × 300 × 5 mm.
- Single screw extruder, type W45 (IMPiB, Torun, Poland), equipped with a screw 45 mm in diameter and an L/D ratio of 30, intended to produce profiles with dimensions of 30 × 30 × 1000 mm.

### 2.3. Sample Preparation

Granulated ABS composites were prepared with the use of a co-rotating twin screw extruder. In the first stage, ABS concentrates containing 70 wt% of each type of GR (GRd or GRm) were produced. In both cases, ABS and GR were introduced into the extruder feed zone using two calibrated metering feeders, which allowed concentrates of strictly defined compositions to be obtained. The temperatures of the particular barrel heating zones and extrusion die-head were 210, 215, 220, 225 °C and 225 °C, respectively. The screw rotation speed was constant at 250 rpm. The plasticizing system was equipped with one non-vacuum degassing at L/D 32. ABS was dried before processing at 80 °C for 4 h. GR was not subjected to drying. The extruded concentrates were granulated directly on the die-head and simultaneously intensively cooled in an air stream. In the second step, the obtained concentrates were diluted to the appropriate amounts of GR in the polymer matrix, and the relevant components were added according to the information presented in Table 1.

The amounts of individual impact modifiers were used according to the recommendations of the respective suppliers. They were usually added in the amount of 3 to 15 wt%. All components were subjected to cold mechanical mixing, and then the homogenized mixture of individual components was dosed using a volume feeder into the extruder hopper feeder. Temperatures, screw rotation speed and granulation method on the die-head were the same as for extrusion of ABS concentrate. The designations and compositions of the extruded granule samples are listed in Table 1. A sample of the pure, non-extruded ABS was used as a reference sample. As a result of the work carried out, 21 types of composites containing shredded WTB were obtained and studied.

**Figure 2:**
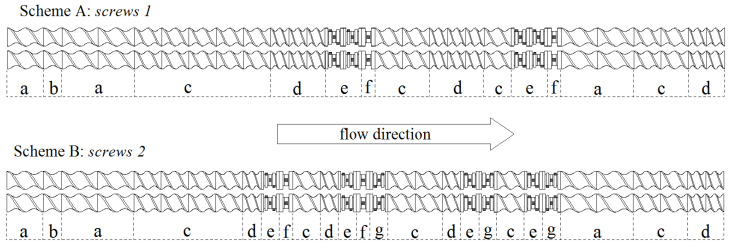
Design of screws of a co-rotating twin screw extruder; Scheme A—*screws 1*; Scheme B—*screws 2* (a—conveying segments of 40-mm length and 40-mm pitch, b—conveying segments of 20-mm length and 40-mm pitch, c—conveying segments of 30-mm length and 30-mm pitch, d—conveying segments of 20-mm length and 20-mm pitch, e—kneading segments of 20-mm length, providing intensive distributive mixing, f—kneading segments of 15-mm length, providing intensive dispersive mixing, and g—reverse kneading segments of 20-mm length, providing intensive distributive mixing).

The extrusion process was carried out with the use of two types of screws, differing in the design of some segments, which led to different ways of plasticizing. Screws of one type (*screws 1*) were of a relatively simple design; they were composed of conveying segments, differing merely in the compression ratio as well as two sections composed of kneading segments (Figure 2, Scheme A). Screws of the second type (*screws 2*) were of a more complex design; they consisted of conveying segments, reverse segments and kneading segments, including elements providing intensive dispersive mixing and intensive distributive mixing (Figure 2, Scheme B).

*Screws 1* were used to carry out the extrusion process under relatively mild conditions, i.e., when the individual components and additives would not be subjected to intense shear forces but only be rapidly transported and slightly mixed. The use of *screws 2* of a more complex geometry was intended to perform extrusion under conditions essentially differing from those concerning the processing with the use of *screws 1*. In this case, it was expected that the processed materials would be subjected to intense shear forces and considerable mixing. The reverse segments of these screws should provide a longer residence time for the materials within the plasticizing system. This should enable more accurate mixing of the components, but it might lead to an increase in the mechanical–thermal degradation of the materials being processed.

During the course of the extrusion, the basic parameters of this process were recorded. They included: extruder motor torque (M) and temperature (T_D_) of the material being processed, measured in the extruder die-head.

The dumbbell- and bar-shaped specimens were prepared according to a relevant standard (PN-EN ISO 527-2:2012) by using an injection molding machine (Battenfeld Plus 35/75, Solingen, Germany). The temperatures of barrel plasticizing zones I and II were 215 and 220 °C, respectively, and that of the injection die-head, 225 °C. The temperature of the injection mold varied over the range of 40–65 °C and the injection pressure, over the range of 130–170 MPa, depending on the composite kind.

The hot-pressing process was carried out at 190 °C. After the temperatures of the heating plates stabilized, a defined amount of composite was introduced into the mold and heated for 5 min. The composite was pressed in two stages; first at 40 bar (5 min) and then at 160 bar (5 min). The cooling time was 2 min. Before the hot-pressing process, the composite granulate was dried at 80 °C for 4 h.

Extrusion of the profile was carried out at temperatures of the barrel heating zones 180–200 °C and extrusion die-head 170 °C. The screw rotation speed was constant at 15 rpm. Prior to processing, the composite was dried at 80 °C for 4 h. A calibrator, a 5 m long cooling bath, a double-track puller and an automatic saw were used to produce the profiles. Finally, meter-long sections of the profile were obtained.

### 2.4. Research Methods

- The surface geometrical structure of the sample fractures and adhesion at the phase boundary was determined by SEM using a secondary electron (SE) detector and an accelerating voltage of 10 kV. Fractures were made during impact tests. A 10-nm thick gold layer was sputtered on all samples to be analyzed by SEM. For that purpose, cathode sputtering apparatus was used, which was equipped with a coating thickness gauge based on a quartz crystal of varying conductivity.
- The mass melt flow rate was measured according to PN-EN ISO 1133:2022 standard (220 °C, 10 kg). Five measurements were performed for each sample in MFR tests.
- The impact strength (a_cN_) was evaluated according to PN-EN ISO 179-1:2010 standard. Ten measurements were performed for each sample in this test.
- The tensile strength (σ_M_), elongation at break (ε_B_) and longitudinal modulus of elasticity (E_t_) were evaluated according to PN-EN ISO 527-1:2020 standard, using an extension rate of 1 (for E_t_) and 50 (for σ_M_, ε_B_) mm/min. The specimens were clamped in the testing machine clamps in such a way that the initial measuring distance (l_o_) was 50 mm. Five measurements were performed for each sample in these tests.
- The flexural strength (σ_fM_) and flexural modulus (E_f_) were measured in a three-point bend test, at a bending deflection rate of 2.0 mm/min. The measurements were carried out in accordance with the PN-EN ISO 178:2010 standard. Five measurements were performed for each sample in these tests.
- The density was measured by pycnometric method, according to PN-EN ISO 1183-3:2003 standard. Five measurements were performed for each sample in the density test.
- The hardness tests were performed by the Shore method according to PN-EN ISO 868:2005 standard. Ten measurements were performed for each sample in these tests.
- The moisture content was determined in accordance with the PN-C 89418 standard. Three measurements were performed for each sample in these tests.
- Determination of ignitability by the oxygen index method was carried out in accordance with the EN ISO 4589-2 standard.
- The resistivity was determined in accordance with the PN-EN 62631-3-1:2016 standard.
- Thermogravimetric analysis (TG) was performed in the temperature range of 25–1000 °C, under a nitrogen atmosphere and at a heating rate of 10 °C/min. A sample of about 26 mg was deposited in an open platinum crucible.
- In addition, due to the essential confidence interval of the measured quantities and small differences between the values of these quantities in the case of some samples, a significance test (Student’s *t*-test or Cochran–Cox test) was performed for the respective two means, assuming a significance level α/2 = 0.05. The Cochran–Cox test was applied only when the hypothesis as regards the equality of two variances was rejected in favor of an alternative hypothesis based on the Fisher–Snedecor test.

In all investigations, the arithmetic mean of individual results was taken as the final result in measured quantities. Furthermore, the studies of density, hardness, moisture content, oxygen index, resistivity and thermal stability were performed only for the selected composite with the most promising properties.

## 3. Results and Discussion

The results of studies are presented in four sections covering different issues. The first (Section 3.1) refers to studies of the processing and properties of the obtained composites, depending on the type of screw shape used. The second (Section 3.2) relates to the study of the effect of impact modifiers mainly on the mechanical properties of composites containing 30 or 50 wt% GRm. The next section (Section 3.3) presents the effects of hot-pressing and extruding the selected composite, as well as shows the final products. The last section (Section 3.4) concerns the investigations on the dust fraction containing GRd in the amount of 10 to 70 wt%.

### 3.1. Studies with the Use of Different Screws

**Figure 3:**
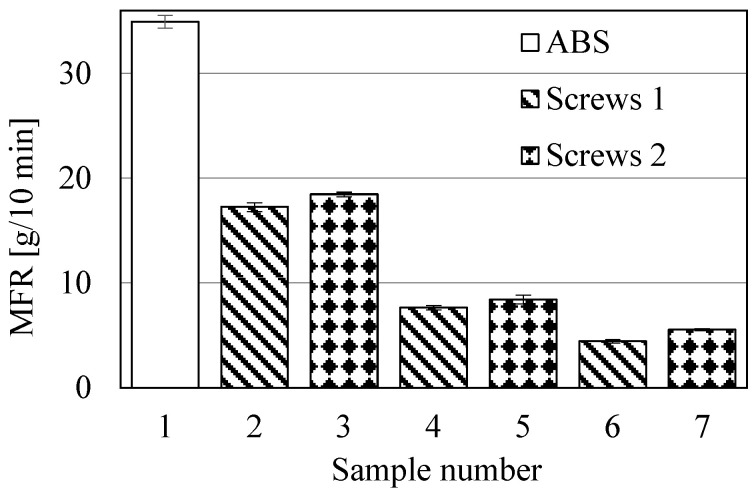
MFR results (samples 2, 4 and 6 were processed with the use of *screws 1*, while samples 3, 5 and 7 were processed using *screws 2*; sample 1 is non-processed ABS).

**Figure 4:**
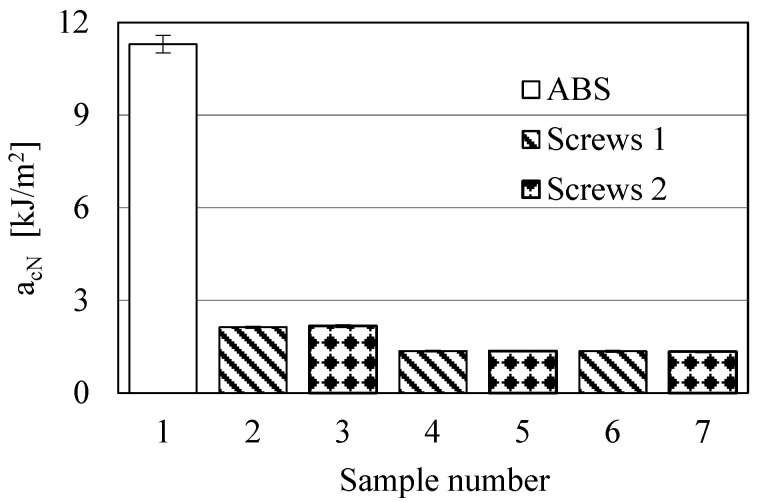
Impact strength results (samples 2, 4 and 6 were processed with the use of *screws 1*, while samples 3, 5 and 7 were processed using *screws 2*; sample 1 is non-processed ABS).

The objective of these studies was to determine the influence of the screw shape (configuration of its segments) on the MFR values, impact strength, structure and processing of ABS composites with GRm content of 30, 50 and 60 wt% (samples 2–7 in Table 1). The results of the MFR and impact strength are shown in Figure 3 and Figure 4, respectively. The MFR and impact strength analyses were deliberately performed because it was expected that the most significant changes could be observed in these two areas.

**Figure 5:**
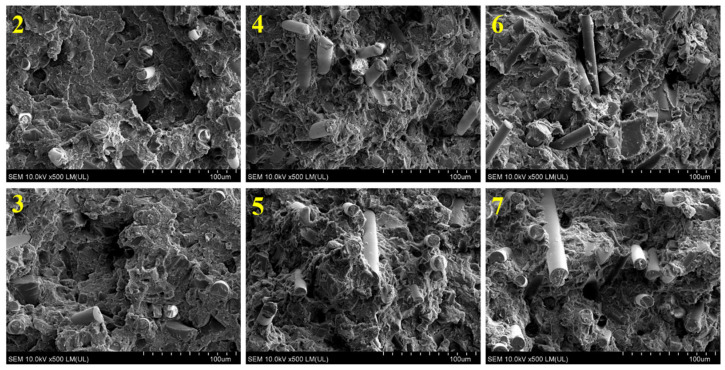
SEM images of composites 2–7 (numbering according to Table 1).

The results shown in Figure 3 indicate a significant decrease in the MFR value of ABS with increasing GRm content. The largest decrease is observed in sample 6: it is 87%. Reducing the MFR value to 4–5 g/10 min is detrimental for the injection molding process of this type of composite, but may prove desirable in other technological processes, such as profile extrusion. In turn, comparing the MFR values of samples processed with both types of screws, it can be concluded that slightly higher values are observed in samples processed with *screws 2.* This indicates greater mechanical–thermal degradation of these samples. It is also worth noting that, based on significance tests, all the results shown in Figure 3 are significantly different from each other. The situation is completely different in the case of the impact test results (Figure 4). Here, the results of impact tests of samples with the same composition, but processed with different screws, do not differ significantly from each other. This indicates that impact strength does not significantly depend on the type of screw shape used. Furthermore, the introduction of as little as 30 wt% GRm into the ABS matrix causes a very large decrease in impact strength value, which is about 82% regardless of the type of screws. Larger amounts of GRm cause a further decrease in impact strength, but it is not so significant. The use of different types of screws also does not affect the structure of the investigated composites, which is similar in all samples. All the fractures are of a brittle character, and the adhesion at the ABS–GRm interface is practically the same in each sample regardless of the type of screws used (Figure 5). Moreover, the adhesion between both components is quite good. Visible craters are not any material defects, but a replica of the other half of a sample fracture.

The significantly decreased impact strength of ABS due to the addition of GRm, regardless of the type of screws used, is a major challenge in the manufacture of such composites. Therefore, the use of additional components, mainly those increasing the dynamic impact strength of ABS/GRm composites, seems to be necessary. On the other hand, from the results presented in this section, it is difficult to clearly state which type of screws is better for ABS/GRm composite production. Only the MFR results suggest that better screws may be those of relatively simple design. The definitive choice of this type of screw (*screws 1*) is mainly supported by the processing parameters of the studied composites. It was observed that the use of *screws 2* caused the occurrence of much higher values of torque (M), especially during the processing of composites with higher filling (over 50%). For example, the M value of sample 7 was on average 3 times higher compared to sample 6. Significantly better processing conditions associated with lower energy consumption (lower extruder motor torque; lower values of the temperature of the material being processed, measured in the extruder die-head, and lower unit power consumption) during the processing of the studied composites with *screws 1*, as well as a lower degree of degradation estimated in the MFR study, indicate that *screws 1* are a more favorable solution in the processing of discussed composites. Therefore, further studies, presented in Section 3.2, were performed exclusively with their participation.

### 3.2. Impact Modification of ABS/GRm Composites

As shown in the previous section, the quantity requiring significant improvement is impact strength. For this purpose, five commercially available impact modifiers (SIS, CPE, ACM, SC and BYK) were used, the specifications of which are presented in Section 2.1. Modifying the ABS/GRm composites with these additives, other mechanical properties were additionally investigated, and mainly such quantities as σ_M_, ε_B_, E_t_, σ_fM_ and E_f_ were measured. The values of MFR and changes in the basic processing parameters, i.e., M and T_D_, were also determined. The investigations were performed for ABS composites containing 30 wt% GRm (samples 8–14) and 50 wt% GRm (samples 15–17). Composites with lower GR content were not studied due to their lack of practical importance; it is important to reuse as much WTB waste as possible. Samples 2 (ABS with 30 wt% GRm) and 4 (ABS with 50 wt% GRm) were reference samples relative to the corresponding samples containing impact modifiers. The results of the above studies are summarized in Table 2 (composites containing 30 wt% GRm) and Table 3 (composites containing 50 wt% GRm).

The data shown in Table 2 and Table 3 indicate several important issues. Firstly, it can be seen that the impact strength of modified composites (samples 8–14 and 15–17) generally improved slightly compared to the impact strength of ABS composites with 30% or 50% GRm. Although in percentage terms, a large improvement in impact strength can be observed (up to 43% in the case of sample 14 or 105% in the case of sample 17), in absolute terms it does not exceed 3 kJ/m^2^, and thus is still small. Moreover, it is almost 4 times lower compared to the impact strength of pure ABS. The tensile strength (σ_M_) of all investigated samples deteriorated compared to reference samples 2 and 4. Nevertheless, it is still quite good, and samples characterized by σ_M_ values above 30 MPa can be used in a wide range of applications, such as for the production of panels or profiles. Analogous changes are observed in the results of σ_fM_. Only the σ_fM_ values of samples 2 and 12 do not differ significantly from each other, as indicated by significance tests. The ε_B_ values of the studied samples are similar regardless of the modifier used and range from 2 to 6%, which is characteristic of this type of engineering composites. The modulus of elasticity (E_t_ and E_f_) increases in most cases (mainly in composites with 30% GRm) as impact modifiers are added. However, their values decrease in composites with 50% GRm content. The E_t_ values of samples 13 and 14 also decrease compared to sample 2.

The modifiers used generally do not worsen the processing conditions, and some even improve them, as evidenced by the M and T_D_ values. In the case of the M quantity, it can be stated that its values do not change or decrease by no more than 36%, for example, in samples 11 or 15, which is a favorable effect. T_D_ values also remain constant. The largest deviations from the reference values, i.e., 235 °C for sample 2 and 238 °C for sample 4, are 2.0 and 1.5%, respectively, which is relatively small. Analyzing all the results together, it was found that the sample with the optimal composition for further studies in the field of hot-pressing of panels or extrusion of the profile is sample 10. It exhibits a relatively low MFR value, and at the same time improved impact strength, increased elastic modulus and quite good tensile strength. The MFR value of this sample (ca. 8 g/10 min) is recommended for both processes. Samples 9 and 11 also show a similar value, but they have a lower tensile strength. Taking into account also the stability of the processing of this type of composite, it seems that it could be a good candidate for practical use in selected applications. Moreover, the ACM-G2 modifier used in this composite is characterized by a much better price–quality ratio compared to other modifiers, which is also of great application importance, especially when similar modification effects are achieved. Based on the results obtained at this stage of the investigation, one more conclusion can be formulated. Improving the mechanical properties of composites based on ABS and appropriately shredded WTB is difficult, especially if the polymer matrix itself is also derived from recycling.

### 3.3. Panels and Profiles Manufactured by Hot-Pressing and Extrusion, Respectively

**Figure 6:**
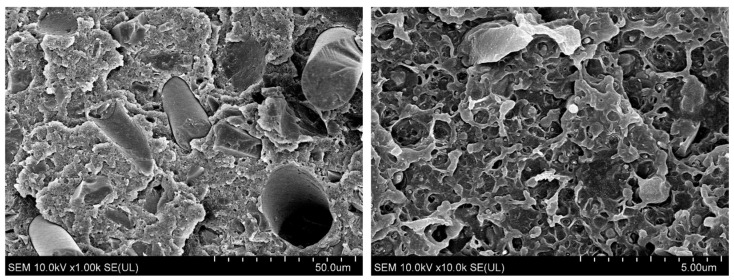
The structure of ABS/GRm/ACM-G2 composite (sample 10).

The composite selected for hot-pressing and extrusion processes (ABS containing 30 wt% GRm and 7 wt% ACM-G2) was subjected to detailed investigations to determine its functional properties. The structure, density (d), moisture absorption (MA), oxygen index (OI), and thermal stability (T_max._) of this composite were analyzed. The resistivity (ρ) and hardness (H) of a 100 mm × 100 mm × 1 mm specimen injected from this composite were also investigated. The results of these studies are presented in Figure 6 and summarized in Table 4.

The composite intended for the production of panels or profiles was characterized by a density close to the theoretical value corresponding to the composition of this composite. In the structure of the composite, both fibers, holes formed by removed fibers, resin particles, as well as modifier particles can be observed. This structure is characteristic of brittle samples. Moreover, at higher magnification, the presence of a small amount of micropores can also be seen. They occur in the polymer matrix forming a spatial network. The presence of micropores may result from the thermal decomposition of some of the volatile components in the impact modifier. According to the producer’s information, these volatile components may be up to 1.5%. Micropores can also result from insufficient drying of the composite before processing, because it absorbs moisture quite easily. This is an unfavorable effect, therefore thorough drying of the composite before further processing is required. The obtained oxygen index value (ca. 21%) of the studied composite is similar to that of the epoxy resin and is only slightly higher than that of pure ABS. It indicates that the prepared composite is characterized by flammability on the border of flammable and self-extinguishing materials. Thus, the WTB fraction used does not influence the change in flammability of the obtained material. The maximum decomposition rate temperature (T_max._) of the composite, which is higher than 300 °C, indicates that the obtained composite exhibits quite good thermal stability. It is also a composite, when injection molded, that is characterized by high hardness and lack of electrical conductivity, making it an excellent dielectric.

**Figure 7:**
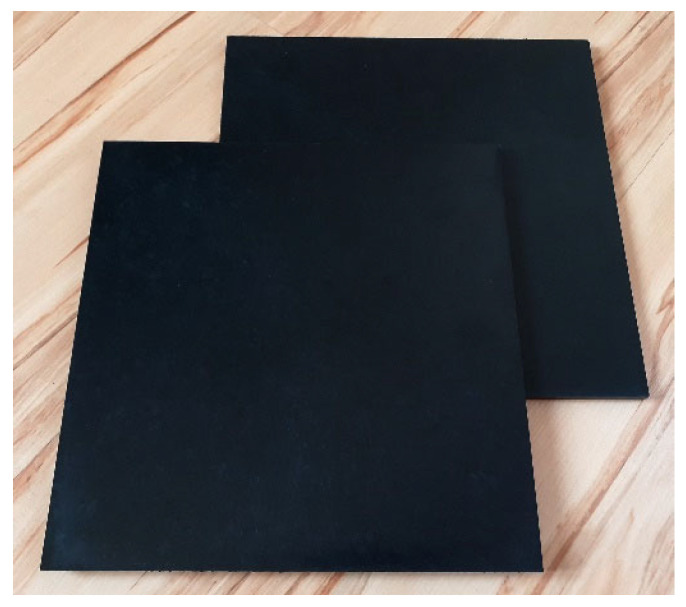
Panels obtained by hot-pressing.

**Figure 8:**
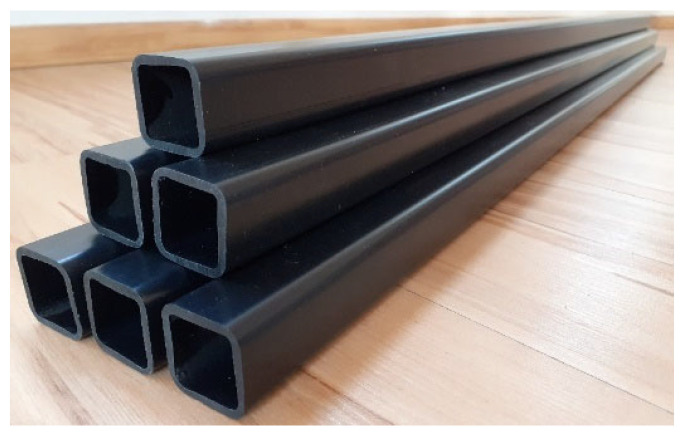
Profiles obtained by extrusion.

During the application tests (hot-pressing and extruding), the developed composite exhibited high processing stability. This made it possible to produce a series of high-quality products in the form of panels (Figure 7) or profiles (Figure 8). These products were characterized by good mechanical properties, e.g., the panels had a flexural strength of 38 MPa and an impact strength of 15 J. Importantly, the products obtained were characterized by very good dimensional parameters, i.e., minimal shrinkage was observed. The panels and profiles that were developed also showed high strength and rigidity and can therefore be used, for example, in the construction industry. Other potential application areas for the developed composites could also include greenhouse frames (horticultural sector), signs (advertising sector) or storage boxes.

### 3.4. Studies of ABS/GRd Composites

**Figure 9:**
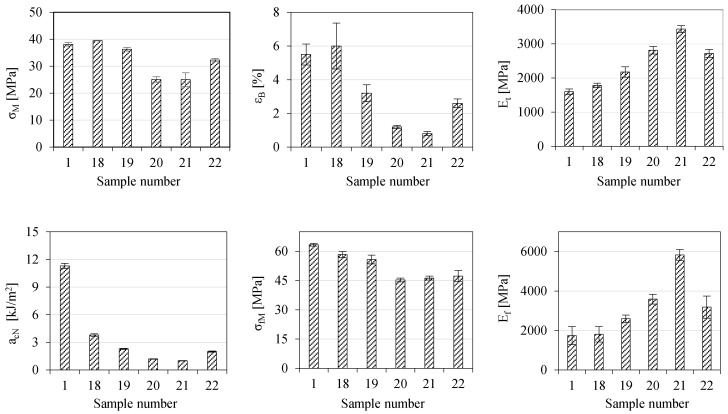
Results of mechanical properties of ABS/GRd composites.

**Figure 10:**
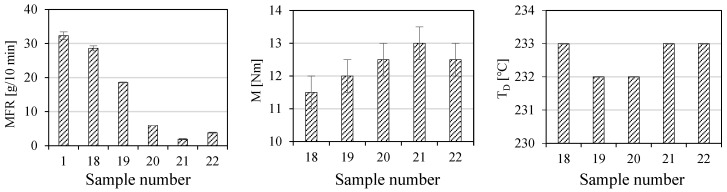
Results of MFR and processing parameters (M and T_D_).

The dust fraction (GRd), which is a waste material generated in significant amounts during the shredding of WTB, should also be properly managed. Therefore, five composites with different GRd contents in the ABS matrix were prepared, one of which also contained the impact modifier SBS (samples 18–22 in Table 1). The results of the mechanical properties (a_cN_, σ_M_, ε_B_, E_t_, σ_fM_ and E_f_) of these composites are shown in Figure 9. The changes in MFR, M and T_D_, are shown in Figure 10.

It follows from Figure 9 that the dusty fraction (GRd) in the smallest amount (10% by mass) does not significantly affect the changes in most of the examined quantities. Only the impact strength significantly decreased, by 66%. A larger amount of GRd (30% by mass) no longer significantly affects only the values of σ_M_. However, it significantly increases the values of both measured moduli, and decreases the values of the other quantities. Moreover, the largest amounts of GRd (50 or 70% by mass) significantly deteriorate the strength of the composite, as well as its impact strength and elongation at break. The greatest deterioration in mechanical properties can be observed in impact strength and elongation at break. The values of both measured quantities, i.e., a_cN_ and ε_B_, which are less than 2 kJ/m^2^ and 2%, respectively, indicate a very brittle material that is subject to destruction under both low-force dynamic impact and small deformation. It is also worth noting that the values of a_cN_, σ_M_, ε_B_, and σ_fM_ of samples containing 50 or 70 wt% GRd are not significantly different from each other. A very large increase is observed only in the values of E_t_ and E_f_, but this is an obvious effect of the stiffening of the sample at such a high content of the fraction containing glass fibers. The deteriorated mechanical properties can be significantly improved by the addition of an SBS modifier (sample 22). As a result of such an action, all measured quantities improved relative to those of sample 20. Moreover, sample 22 also became less stiff. It follows that in the production of this type of composite, the addition of flexible SBS-type modifiers is beneficial.

It follows from Figure 10 that MFR values decrease linearly as the GRd content in the ABS matrix increases. The maximum decrease in MFR values is as high as 94%. Changes in M are less visible, but in general, the trend is increasing as the amount of GRd increases. In this case, however, it should be said that not all results are significantly different from each other. Significance tests show that the changes are significantly different if the composites differ in GRd content by at least 40%. T_D_ values, in contrast, practically do not change, and differences of 1 °C are within the measurement errors of the temperature sensor. Moreover, the MFR value of sample 20 decreases by 36% after adding an impact modifier to it. However, M and T_D_ values do not change significantly in the presence of this modifier.

The use of a dusty fraction (GRd) in the manufacture of ABS composites causes slightly different effects than when GRm fraction is used. Although the MFR and impact strength values of composites containing both types of GR are similar, the mechanical properties of these composites in static tension and static bending are significantly different. They are usually better when GRm is used. This refers to such quantities as σ_M_, σ_fM_, E_t_, and ε_B_, but only for composites containing 50 wt% GRm. Composites containing GRd also exhibit lower M and T_D_ values compared to those containing GRm. These changes are mainly due to the degree of fragmentation of the WTB and the presence of glass fibers of different lengths in each fraction. In the case of the GRd fraction, the fibers are considerably damaged and short, and therefore exhibit much worse reinforcing functions compared to the GRm fraction.

## 4. Conclusions

The ABS modified with suitable fractions of shredded wind turbine blades shows significant changes in the mechanical properties. Most mechanical parameters generally deteriorate. This is especially true for impact strength, the values of which decrease to the greatest extent, even when the smallest amounts of GRm are added. Therefore, this quantity requires the greatest effort in modifying ABS/GRm composites. Of the group of commercial impact modifiers tested, the grafted copolymer of light-chlorinated HDPE and acrylate (ACM-G2) showed the most promising properties. With the use of this modifier at 7% by mass, an ABS/GRm composite with the best material properties and good processing parameters can be obtained. It also has a good price–quality ratio. A composite containing 30 wt% GRm and 7 wt% ACM-G2 can be hot-pressed into good-quality panels that can find a wide range of applications, including in the construction industry. The composite is also suitable for profile extrusion, mainly due to the significant reduction in viscosity. The composites containing a dusty fraction (GRd), which were additionally studied, showed slightly deteriorated properties compared to the corresponding composites containing a GRm fraction. In their case, it is also necessary to use suitable modifiers. One such modifier could be the styrene-butadiene-styrene copolymer (SBS). The studies performed in the field of mechanical recycling have shown the possibility of using shredded WTB in the production of thermoplastic matrix composites. This is one of the simpler forms of recycling, but with great possibilities and it uses commonly available polymer processing machinery and equipment. Taking also into account the fact that the matrix in our study was ABS regranulate obtained from the recycling process, practically the whole WTB recycling cycle was based on secondary raw materials. This is a favorable approach, and the attempt to use WTB in a new product based on other recycled raw materials also seems to be as beneficial as possible, especially for the environment. The development of such materials is a direction in which further studies should be conducted. Further studies should also be carried out to improve interfacial adhesion, mechanical properties and cost reduction in the manufacture of such composites.

## Figures and Tables

**Table 1:** Symbols and compositions (wt%) of studied samples.

Research Stage	Sample Number	Screw Type	ABS	GRm	GRd	SBS	SIS	CPE	ACM	SC	BYK
All	1	–	100								
Stage I (Section 3.1)	2	1	70	30							
	3	2	70	30							
	4	1	50	50							
	5	2	50	50							
	6	1	40	60							
	7	2	40	60							
Stage II (Section 3.2 and Section 3.3)	8	1	60	30				10			
	9	1	65	30					5		
	10	1	63	30					7		
	11	1	60	30					10		
	12	1	69.5	30							0.5
	13	1	60	30						10	
	14	1	55	30						15	
	15	1	40	50			10				
	16	1	33	50					7	10	
	17	1	37	50			3		5	5	
Stage III (Section 3.4)	18	1	90		10						
	19	1	70		30						
	20	1	50		50						
	21	1	30		70						
	22	1	40		50	10					

**Table 2:** Results of selected properties of ABS/GRm composites containing 30 wt% GRm and impact modifiers.

	Sample Number	a_cN_ [kJ/m^2^]	σ_M_ [MPa]	ε_B_ [%]	E_t_ [MPa]	σ_fM_ [MPa]	E_f_ [MPa]	MFR [g/10 min]	M [Nm]	T_D_ [°C]
**ABS**	1	11.3 ± 0.4	42.3 ± 0.9	5.5 ± 0.5	1599 ± 62	63.4 ± 0.6	1742 ± 369	32.6 ± 0.9	-	-
**ABS + 30 wt% GRm + additives**	2	2.1 ± 0.1	43.5 ± 1.1	3.4 ± 0.83	2440 ± 86	62.9 ± 1.4	2302 ± 63	17.3 ± 0.4	24	235
	8	2.6 ± 0.1	26.0 ± 2.4	2.3 ± 0.30	2222 ± 19	48.5 ± 0.3	2429 ± 610	0.7 ± 0.1	22–23	232
	9	2.4 ± 0.1	30.3 ± 3.1	2.2 ± 0.45	2511 ± 28	53.6 ± 0.7	3825 ± 56	8.5 ± 0.4	23–24	234
	10	2.4 ± 0.2	33.2 ± 1.6	2.6 ± 0.4	2569 ± 55	53.3 ± 0.4	3493 ± 78	8.2 ± 0.5	22	235
	11	2.7 ± 0.2	28.8 ± 0.6	2.0 ± 0.07	2482 ± 41	49.8 ± 0.3	3456 ± 48	7.8 ± 0.1	16–18	239–240
	12	2.0 ± 0.2	35.5 ± 2.6	2.0 ± 0.2	2685 ± 50	61.9 ± 0.4	4034 ± 30	14.4 ± 0.3	19	240
	13	2.9 ± 0.2	38.5 ± 0.2	4.9 ± 0.4	2132 ± 23	57.0 ± 0.5	2964 ± 61	12.9 ± 0.2	20–21	238–239
	14	3.0 ± 0.2	34.5 ± 0.2	6.5 ± 0.3	1997 ± 43	51.6 ± 0.7	2593 ± 36	12.7 ± 0.4	18–19	235

**Table 3:** Results of selected properties of ABS/GRm composites containing 50 wt% GRm and impact modifiers.

	Sample Number	a_cN_ [kJ/m^2^]	σ_M_ [MPa]	ε_B_ [%]	E_t_ [MPa]	σ_fM_ [MPa]	E_f_ [MPa]	MFR [g/10 min]	M [Nm]	T_D_ [°C]
**ABS**	1	11.3 ± 0.4	42.3 ± 0.9	5.5 ± 0.5	1599 ± 62	63.4 ± 0.6	1742 ± 369	32.6 ± 0.9	-	-
**ABS + 50 wt% GRm + additives**	4	1.36 ± 0.1	45.3 ± 3.1	2.6 ± 0.5	3202 ± 101	62.4 ± 2.2	4081 ± 602	7.6 ± 0.2	22	238
	15	2.3 ± 0.1	24.9 ± 0.1	2.9 ± 0.1	2358 ± 63	38.4 ± 1.0	3282 ± 178	22.0 ± 0.2	14	240
	16	2.6 ± 0.1	33.2 ± 0.2	4.5 ± 0.5	2330 ± 74	49.6 ± 1.8	3178 ± 549	2.33 ± 0.2	18	242
	17	2.8 ± 0.1	30.1 ± 1.5	2.4 ± 0.2	2532 ± 34	47.4 ± 2.1	3027 ± 442	4.53 ± 0.1	19–20	235

**Table 4:** Functional properties of ABS/GRm composite containing 7 wt% ACM-G2.

Parameter	d [g/cm^3^]	MA [%]	OI [%]	T_max._ [°C]	H [Shore D]	ρ [Ωcm]
Result	1.19	0.383	20.7	327.4	68	3.14 × 10^15^

## Data Availability

The original contributions presented in the study are included in the article, further inquiries can be directed to the corresponding author.
